# Season of birth is different in Inuit suicide victims born into Traditional than into Modern Lifestyle: a register study from Greenland

**DOI:** 10.1186/s12888-015-0506-x

**Published:** 2015-07-04

**Authors:** Karin S Björkstén, Peter Bjerregaard

**Affiliations:** 1 Psychiatry South Stockholm, Ledning & Administration, Box 5040, SE-121 05 Johanneshov, Sweden; 2 Department of Neurobiology, Care Sciences and Society, Karolinska Institutet, Stockholm, Sweden; 3 National Institute of Public Health, University of Southern Denmark, Øster, Farimagsgade 5A, 2nd floor, DK-1353 Copenhagen K, Denmark

**Keywords:** Suicide, Season of birth, Inuit, Greenland, Traditional Lifestyle, Light, Register study

## Abstract

**Background:**

There is growing evidence that living conditions at birth play a role in medical conditions later in life. Population-based studies from the Northern Hemisphere have shown that persons born in the spring or summer are at greater risk of committing suicide. A statistical correlation with light availability at birth has been observed in past research, but the cause remains unknown. Greenland is one of the most extreme of natural human habitats with regard to seasonal changes in light. The combination of rapid social changes and reliable population statistics offers a unique opportunity to make comparisons between persons born into a Traditional Lifestyle and those born into a Modern Lifestyle. The aim of this work was to assess whether season of birth differed between suicide victims born into an old or into a modern lifestyle.

**Methods:**

Official population and mortality registers were used. Suicide victims born (1903–1950) into the Traditional Lifestyle were compared with those born into the Modern Lifestyle (1961–1980). Rayleigh’s test for circular distributions was used to assess the season of birth in suicide victims. Data regarding season of birth in the general population were collected.

**Results:**

Persons born in March-June in the Traditional Lifestyle were much less likely to commit suicide than those born during other periods of the year. This is contrary to the findings of other studies. The seasonal differences had disappeared for those born into the Modern Lifestyle. The suicide rate increased from very low rates to about 140 suicides/100 000 person-years in the 1980s.

**Conclusions:**

The reason behind a variation in season of birth in suicide victims born into the old lifestyle is unknown. It is also unknown why the seasonal difference had disappeared with modern lifestyle. Possible influence of artificial light, nutrition, microbiota and seasonal infections are discussed. The underlying causes behind suicides may be different in traditional and modern Greenland.

## Background

There is growing evidence that season of birth plays a role in a variety of conditions such as suicide [1–5], schizophrenia [6], bipolar disorder [7], cardiovascular disorders [8], allergy [9] and longevity [10]. It has been proposed that nutrition, seasonal infections, gut flora and photoperiod also contribute. Studies of season of birth are mostly based on computerised population data from developed countries. There are few remaining places where the population until recently lived in a traditional lifestyle and where reliable population data are available. Greenland is one of them. This study addresses the impact of the season of birth in suicide victims born in Greenland.

### Season of birth in suicide victims

A few studies have shown that the risk for suicide varies with season of birth and light has been proposed as a causative factor [11]. A study covering suicide victims (*n* = 9073; dead 1979–88) at several locations from Pennsylvania 40°N to Alaska 61°N found that hours of sunlight available at the time of birth correlated with the numbers of suicide victims born at that time [3]. The differences were strongest in Alaska Natives living at high latitudes, where the seasonal variations in daylight were greatest. The risk of suicide was higher among Hungarians born in spring and summer with the highest risk among those born in July (*n* = 78 740; born 1930 and later; dead 1970–2008) [2]. In England and Wales, the risk of suicide was highest among those born between April and June (*n* = 26 915; born 1955–1966; dead 1979–2001) [5]. A study conducted in northern Sweden suggests that suicide methods among suicide victims who had not been in contact with the psychiatric services (*n* = 693; dead 1961–1980) differed as a function of month of birth [1]. Death by hanging was the most prevalent method among suicide victims born between February and April whereas death by poisoning or use of petrol gas was the most prevalent method among those born in September. In northern Finland, suicide victims with schizophrenia (*n* = 228; dead 1989–2010) were more often born in the summer than suicide victims without a hospital-treated mental disorder (*n* = 1434; dead 1989–2010) [6]. Results from a small study suggested an excess of August births in suicide victims aged 55 and older in North Cheshire, UK. [4]. However, other studies in the US [12–14] and in Western Australia [15] have been unable to find such association.

### Light in relation to melatonin, serotonin and biological rhythms

Light regulates the circadian rhythm by synchronising the circadian pacemaker in the suprachiasmatic nucleus by acute inhibition of melatonin production in mammals [16]. The duration of the melatonin signal informs the body of the season and promotes the adequate physiology for the season. The daily increasing light in the spring phase advances the biological rhythms and the declining light after the summer equinox phase delays it. Circadian gene polymorphism is associated with affective disorder [17] that is highly associated with suicide. Exposure to light releases serotonin in the human brain [18]. A remarkably consistent association between low concentrations of the serotonin metabolite CSF-5-HIAA and suicidal behaviour has been demonstrated [19]. Monoamine metabolites in the CSF vary with season of birth in Swedish psychiatric patients [20] and in febrile infants aged up to 3 months born in St Louis, USA [21].

### Season of death in suicide victims

We have previously reported that suicides in Greenland are almost exclusively violent and peak in the summer [22] and that there is a strong accentuation of the seasonal concentration in the north with several months of constant sun [23]. Numerous studies worldwide have found peaks for death by suicide in the spring or summer and some studies found violent suicides to be more seasonal than non-violent ones [24–26]. A triggering role of sunshine has been proposed [27]. There are a growing number of studies showing that the season of birth has an impact on the risk of suicide and light has again been proposed to play a role. Since Greenland is one the most extreme natural human habitats regarding annual variation in light, we assumed that this variation would also be found in Greenland. We analysed our database but did not find any seasonal difference in the births of the suicide victims. Since the living conditions in Greenland have changed totally during the latter part of the 20th century, we decided to analyse the material with regard to the living conditions at the time of birth.

Little is known about the impact of the season of birth in people living in preindustrial societies. The majority of suicide victims in published studies were modern people in developed countries. Due to the extremely rapid change in lifestyle combined with high quality population statistics, Greenland offers a unique opportunity to make comparisons between suicide victims born under traditional lifestyle conditions and those born into a Modern Lifestyle.

### Greenland – a giant leap from traditional to modern lifestyle

Greenland, the world’s largest island, is located between latitudes 59°40′ and 83°35′N. The majority of the population lives along the coast in the province of West Greenland. The climate is arctic to subarctic with cool winters and cold summers. The mean temperature does not normally exceed 10 °C, but global warming is changing the climate in modern Greenland. Daylight varies widely around the year and dependent on latitude. Greenland is split by the Arctic Circle, where the north has midnight sun in the summer and all day darkness in the winter.

The native population is Inuit. Greenland became a Danish colony in 1721 and was Christianised in the 18th century. The Lutheran-Evangelical church played an important role.

Until the 1950s Greenland was by and large an isolated and traditionally Inuit society where most people lived in small villages and subsisted on small-scale hunting and fishing. Visitors needed special permission from the Danish authorities and mainly Danish officials visited Greenland. In the old Inuit society, life span was short and few reached old age. Tuberculosis, accidents and pneumonia were key causes of death [28]. In 1901–1930 in West Greenland, the average age of death was 26.2 years. Life expectancy has increased to 58.4 years for men and 67.5 years for women born in Greenland 1977–1981.

Suicide was considered an unusual event and the suicide rate was estimated at 3/100 000 person-years during 1901–1930 by Dr A. Bertelsen, who spent a lifetime collecting health data in Greenland [28]. The suicide rate increased rapidly in the 1980s to reach a maximum of 128.4/100 000 person-years in 1987, an extremely high figure [23].

Nutrition in traditional Greenland was based on marine mammals, fish, birds, eggs, land animals and some plants and berries [29]. This food was nutrient-dense, rich in protein and fat, particularly omega-3 fatty acids and low in carbohydrates. Traditional preparation involves placing meat and fat tissues into skin bags, which are sewn shut and aged for weeks or months under rocks or buried under gravel. Meat was often consumed directly without prior heating. In the winter, mainly stored food was available and food poisoning including botulism was a key cause of death. Starvation occurred. In the spring, the increased daylight made hunting possible and fresh food would become available. Fresh food has different microbiota than that of stored food. This made spring a favourable time from a nutritional point of view in contrast to temperate agricultural countries, when the autumn is harvest time with more food available than in the spring and summer.

Unlike Denmark, Greenland was not occupied by Germany during World War II. The major trading partner became the US, which was allowed to build several air bases. After the war, the prewar status of Greenland as a Danish colony continued for some years. Starting in 1950, the Danish government invested considerable amounts of money to improve living conditions and make Greenland take a giant leap into the modern world. Industries and modern houses were built and a large part of the population moved from the traditional small settlements to the towns. Modern kitchens and imported food changed the eating habits. Schools and medical care were made available to everyone. Goods that were new to the Greenlanders were imported. After having used traditional oil lamps giving a mild, yellow light, kerosene lamps quickly became popular and electrical light was introduced little by little even in remote places. The new light sources changed indoor light and made light available around the clock. In 1953, the constitution was changed and Greenland became a part of Denmark. Today, Greenland is a semi-autonomous modern country within the Kingdom of Denmark.

## The aim of this work

The aim of this work was to assess whether there was a variation in season of birth in suicide victims born in Greenland into a Traditional Lifestyle and in those born into a Modern Lifestyle.

## Methods

### Collection of demographic data and causes of death in Greenland

Since Greenland was Christianised in the 18th century, The Danish clergymen were responsible for registering births and deaths in the church books. They usually did this with meticulous care according to the Scandinavian tradition. Since suicide was not acceptable to the Lutheran-Evangelical church and had implications for the funeral, it was important to establish whether a suicide had taken place. Many of the church books have been saved and can still be read. Dr A. Bertelsen collected and published detailed demographic information about the population of Greenland from the church books for the decades around 1900 [28].

The Chief Medical Officer in Greenland [30] published annual cause of death reports in 1951–1967. Suicide data from 1934 to 1950 were unavailable in printed sources.

From 1968, all deaths in Greenland were collected systematically and registered according to the WHO International Classification of Diseases 8th edition (ICD-8) for 1968–1993 and from 1994 according to the 10th edition (ICD-10). Those data are available in computerised registers. The police and the local doctor have to determine the cause of death in all cases of unnatural death. The local doctor has access to all patient records. Autopsies have been performed in 3.2 % of the cases [31].

### Suicides

This study included only cases that were classified as suicides, not uncertain cases such as accidents. Intentional self-poisoning (ICD-10 X60-65) (ICD-8 E950,3 - 950,9 and E951,1) and freezing to death (coded as “Suicide, unspecified method” ICD-8 E958,0) were classified as non-violent suicides. Hanging (ICD-10 X70; ICD-8 E953,0), Drowning (ICD-10 X71; ICD-8 E954,0), Shooting (ICD-10 X72-74; ICD-8 E9 550), Cutting, piercing (ICD-10 X78; ICD-8 E956,0) and Jumping (ICD-10 X80; ICD-8 E957,0) were considered as violent suicides. In cases of “other specified methods” (ICD-10 X830), unspecified methods (ICD-8 E958,0), Late effect of suicide (ICD-8 E959,0), the diagnosis in the second and third position was used to determine whether the method of suicide was violent or not.

### Cohorts

Suicide victims born 1903–1950 (*n* = 274) were selected to represent those born into a Traditional Lifestyle and those born 1961–1980 (*n* = 763) were selected to represent those born into a Modern Lifestyle. Persons born after 1980 were excluded to avoid a dominance of very young subjects. Those born 1951–1960 (*n* = 317) were assigned to the Transition period.

The selection of the cohorts was a delicate task. The basis for the selection was to elucidate when the majority of the populations lived in a Traditional Lifestyle with little influence of modern life. We decided that those born up until 1950 could be considered to be born into a Traditional Lifestyle. Since the life of a country does not change overnight, we decided to exclude a period of transition, 1951–1960, from the Traditional and Modern Lifestyle cohorts. This was a decade of intense changes in the society and migration from settlements to towns. Persons born 1961–1980 were selected to represent those born into a Modern Lifestyle.

### Assessing seasonal variation of birth in suicide victims

This study of season of birth in suicide victims covers all inhabitants born in Greenland before 1981 who died by suicide in Greenland during the 40-year period 1968–2007. A few suicides that occurred before 1968 in the Traditional Lifestyle cohort were therefore not included in the calculations of seasonality, however they are shown in tables. Detailed official computerised registers using exact dates of birth were used. Seasonal variation was assessed by Rayleigh’s test for circular distributions [32] in each cohort separately. Due to the larger age variation in the Old Lifestyle cohort, the seasonal variation of Old Lifestyle cohort was also assesed after splitting the cohort in cases younger that 47 years and older than 46 years of age. Cases in each cohort were pooled and the exact dates were used for calculations of seasonality. The results of the calculations of Rayleigh’s test are expressed by Rayleigh’s “r” (which varies inversely with the circular dispersion of the data) and by Rayleigh’s “Z” (= n x r^2^) that can be used for testing the null hypothesis of no population mean direction and the *p* value. Calculations and graphs were made in the computer programs Statistica version 9 and Excel version 2000.

### Adjusting the seasonal variation of suicide births in relation to the seasonal variation in all births

Births follow seasonal patterns in most species including humans. It is therefore necessary to adjust the finding of a seasonal birth pattern for one condition to the births in the general population. After performing the Rayleigh’s test for circular distributions using the exact dates of birth, the suicides from all years of the Traditional and Modern Lifestyle cohorts were pooled for each month and compared with representative birth data for that cohort.

For the Traditional Life Style cohort, monthly birth data in the general population from the years 1901–1930 [28] and annual birth statistics for 1903–1950 [33] were available. Since monthly birth data were available only for 1901–30, the monthly average number of births for this period was calculated and then multiplied by 31 920 (the total number of registered births during 1903–1950) and divided by 25 757 (the total number of registered births during 1903–1930).

For the Modern Lifestyle cohort, birth rates from 1961 to 1980 extracted from Statistical Yearbooks were pooled. Monthly birth rates were available and the data did not need further adjustments.

For the Traditional and Modern Lifestyle cohorts, the number of suicide cases born each month were pooled and divided by the number of years under observation for suicides. Thereafter, suicide births were divided by general births for each month and multiplied by 1 000 giving the suicide rate per 1 000 births. This suicide rate per 1 000 births is not to be confused with the commonly shown suicide rate per 100 000 person-years. The average of this suicide rate for the nadir months March-June and for the remaining 8 months July-February was calculated.

### Calculation of suicide rate per 100 000 person-years

For periods without computerised registers, the suicide rate per 100 000 person-years for 1890–1930 and 1924–1933 was collected from Bertelsen’s classical work [28]. Data from 1934 to 1950 were unavailable from printed sources. The number of suicides during 1951–1967 were extracted manually from the annual reports of the Chief Medical Officer in Greenland [30]. For calculation of the suicide rate per 100 000 person-years, the population of 1951 was selected to represent the population of 1951–1954 and the population of 1964 to represent the population of 1960–1967. For each of the years 1968–2007, official computerised registers were used to calculate the suicide rate per 100 000 person-years.

### Inuit ethnicity

In spite of the increasing number of Danish residents in Greenland after 1960, place of birth remains a valid proxy for ethnicity for the group of adults living in Greenland. In October 1930, there were 413 Europeans and 16488 Greenlander living in Greenland. In the census years 1976 and 1982, 17 % of the population were born in Denmark. Those include both ethnic Greenlanders and ethnic Danes. In 2006, 1 % of the population was comprised of citizens of other countries than Denmark.

### Ethical approval

Ethical approval was obtained from the Commission for Scientific Research in Greenland. The authors declare that there is no conflict of interest.

## Results

### The suicide rate per 100 000 person-years

**Fig. 1:**
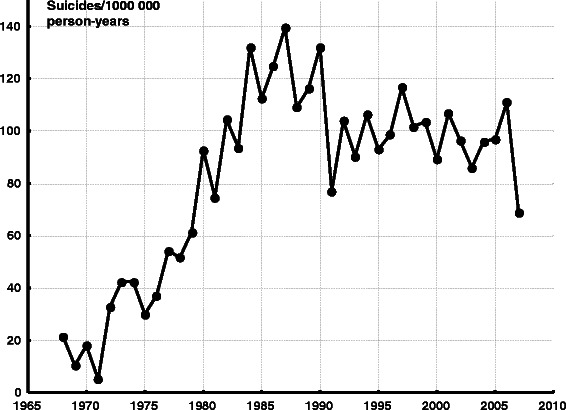
Suicides per 100 000 person-years for all persons born in Greenland plotted against year 1968–2007

The suicide rate per 100 000 person-years for the whole population born in Greenland is shown in Table 1 for the years 1890–1967 (non-computerised sources) and in Fig. 1 for the years 1968–2007 (computerised sources). Data from 1934 to 1950 were not available. During the first half of the 20th century, there were less than 5 suicides/100 000 person-years, however the suicide rate increased to about 14/100 000 person-years in the early 1960s. Around 1970, the suicide rate was still low, but increased to a maximum of 140 suicides/100 000 person-years in 1987 for those born in Greenland. The suicide rate levelled off in the 1990s but is still very high in an international perspective.

**Table 1:** Suicides extracted from non-computerised sources for the years 1890–1967

Time period	Population	Suicides	Suicides/100 000 person-years
1890–1930	not stated	14	4
1924–1933	16 488 (1930)	4	4–5
1934–1950	20 939 (1946)	not available	not available
1951–1954	22 581 (1951)	1	1.1
1955–1959	25 234 (1956)	0	0
1960–1967	33 406 (1964)	38	14.2

The number of suicides extracted from non-computerised sources, the population of the years selected for calculations and the rate of suicides/100 000 person-years for the years 1890–1967. The suicide rate for 1890–1930 and 1924–1933 is from West Greenland

### Seasonal variation in suicide births and suicide rate per 1 000 births

A total of 1354 persons born in Greenland before 1981 died as a result of suicide in Greenland during the 40-year period 1968–2007. Suicides were almost exclusively violent (*n* = 1293; 95.6 %). Violent methods were: hanging (*n* = 657; 48.5 %), shooting (*n* = 537; 39.7 %), drowning (*n* = 51; 3.8 %), jumping from heights (*n* = 22; 1.6 %), cutting or piercing (*n* = 18; 1.3 %). Unspecified violent methods amounted to (*n* = 8) and 61 (4.5 %) were non-violent suicides: poisoning (*n* = 60; 4.4 %) and freezing to death (*n* = 1). Violent methods were used by 98 % (*n* = 1057) of the men and by 86 % (*n* = 236) of the women.

Age and gender for subjects born in Traditional, Transition and Modern lifestyle cohorts are shown in Table 2. There were 274 persons in the Traditional Lifestyle cohort, 317 persons in the Transition Lifestyle cohort and there were 763 persons in the Modern Lifestyle cohort. About 80 % were men and 20 % were women in each cohort. Suicide victims in the Traditional Lifestyle cohort were older compared to the Transition and Modern Lifestyle cohorts.

**Table 2:** Shows age and gender for subjects born into the Traditional, Transition and Modern Lifestyle cohorts who died by suicide 1968–2007

Birth cohort	All			Men			Women		
	n	Years of Age	Years of Age	n	Years of Age	Years of Age	n	Years of Age	Years of Age
		Median (Range)	Mean ± SD		Median (Range)	Mean ± SD		Median (Range)	Mean ± SD
All	1354	27 (11–84)	30.2 ± 12.1	1080	26 (11–84)	30.0 ± 12.1	274	28 (14–80)	31.0 ± 12.4
Traditional 1903–1950	274	44 (17–84)	45.6 ± 13.4	218	44 (17–84)	45.4 ± 13.3	56	46 (22–80)	46.4 ± 13.8
Transition 1951–1960	317	29 (14–53)	30.5 ± 9.4	255	29 (15–53)	30.4 ± 9.3	62	29 (14–52)	30.8 ± 9.5
Modern 1961–1980	763	23 (11–46)	24.6 ± 6.5	607	23 (11–44)	24.3 ± 6.3	156	23 (11–44)	25.6 ± 7.3

**Fig. 2:**
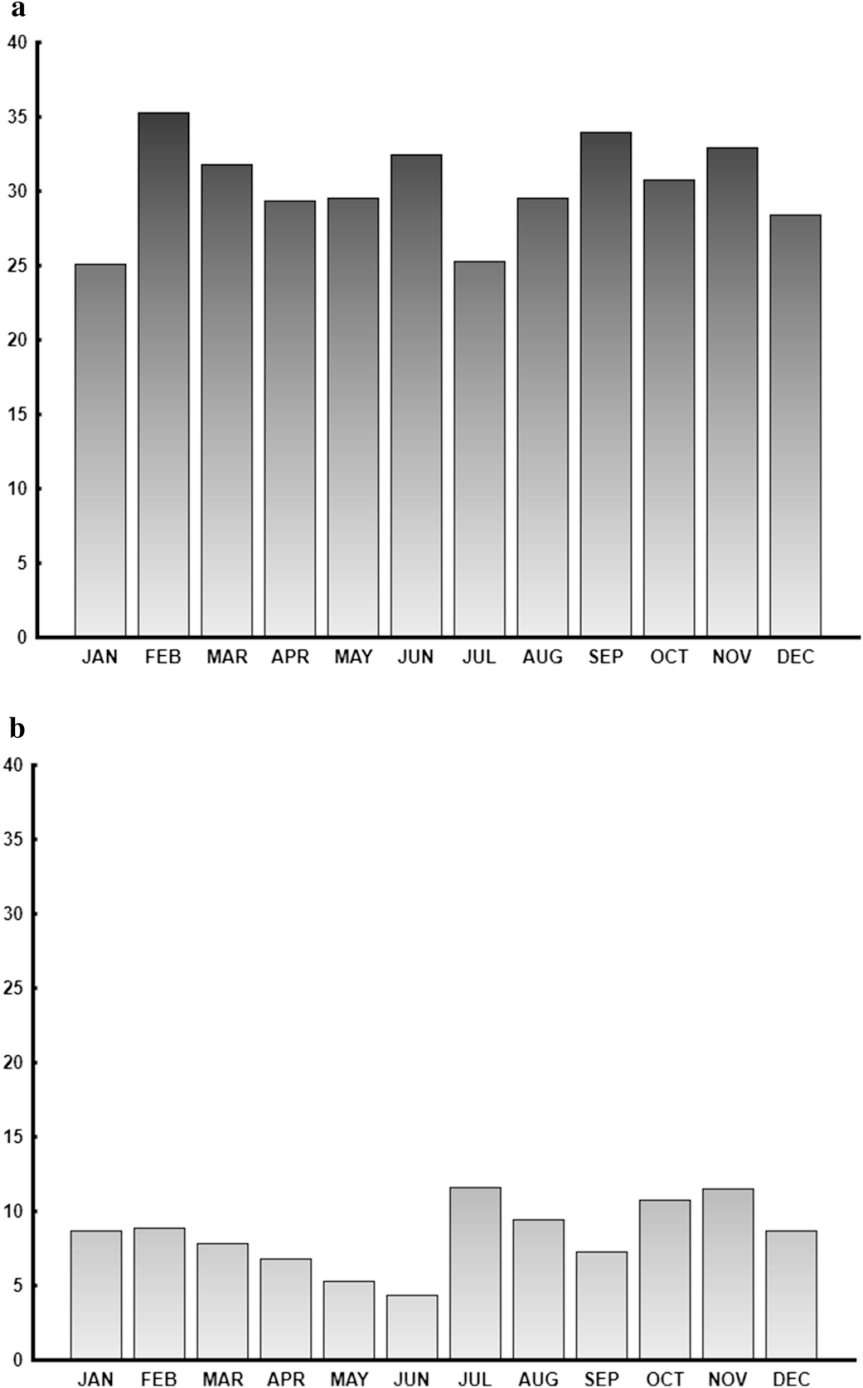
Suicides per 1 000 births for the Traditional and the Modern Lifestyle cohorts. **a** (lower) Suicides per 1 000 births for the Traditional Lifestyle cohort, *i.e.* those born 1903–1950 who died by suicide 1968–2007 shown for each month of the year. **b** (upper) Suicides per 1 000 births for the Modern Lifestyle cohort, *i.e.* those born 1961–1980 who died by suicide 1968–2007 shown for each month of the year

In the Traditional Lifestyle cohort, there was a fairly constant number of suicide victims born every month from July to February and then a decrease until a nadir in June and a sharp rise in July. The variation was highly significant (*n* = 274; r = 0.17; Z = 8.11; *p* < <0.001). When splitting the cohort into a younger (Median 37: Range 17–46 years of age; *n* = 151) and an older (Median 56; Range 47–84 years of age; *n* = 123) subgroup, the seasonal variation was still significant (younger: *n* = 151; r = 0.17; Z = 4.15; *p* < 0.01) (older; *n* = 123; r = 0.20; Z = 4.67; *p* < 0.02). No further calculations were made for the subgroups. The seasonal pattern remained the same after adjusting for births in the population. Figure 2a demonstrates the suicide rate per 1000 births for the Traditional Lifestyle cohort for each month and Table 3 shows the average number of general births, suicide births and the suicide rate per 1000 births for each month during 1903–1950 in the Traditional Lifestyle cohort. The average suicide rate was 10.56 suicides per 1 000 births. For those born in March-June, the average suicide rate was 7.50 and for those born in July-February the rate was 12.09.

**Table 3:** The monthly suicide rate per 1 000 births for the Traditional and Modern Lifestyle cohorts

	Traditional Life-Style Births			Modern Life-Style Births		
Month	Average number of births 1903–1950	Average number of suicide births 1903–1950	Suicide rate per 1 000 births	Average number of births 1961–1980	Average number of suicide births 1961–1980	Suicide rate per 1 000 births
Jan	62.6	0.54	8.7	107.4	2.7	25.1
Feb	58.7	0.52	8.9	99.2	3.5	35.3
Mar	53.2	0.42	7.8	110.2	3.5	31.8
Apr	49.3	0.33	6.8	104.1	3.1	29.3
May	47.1	0.25	5.3	103.2	3.1	29.6
Jun	53.2	0.23	4.3	104.7	3.4	32.5
Jul	53.7	0.63	11.6	106.9	2.7	25.3
Aug	55.4	0.52	9.4	110.2	3.3	29.5
Sep	62.6	0.46	7.3	105.9	3.6	34.0
Oct	56.0	0.60	10.8	102.3	3.2	30.8
Nov	56.0	0.65	11.5	101.8	3.4	32.9
Dec	57.1	0.56	8.7	102.2	2.9	28.4
ALL YEAR	664.8	5.71	8.5	1257.9	38.2	30.4

Table 3 show the average number of births, suicide births and the suicide rate per 1000 births for each month during 1903–1950 in the Traditional Lifestyle cohort. This cohort was defined as those born in Greenland from 1903 to 1950 who died by suicide during 1968–2007. Table 3 also shows the average number of births during 1961–1980, the average number of suicide births during 1961–1980 and the suicide rate per 1000 births for each month in the Modern Lifestyle cohort. This cohort was defined as those born in Greenland from 1961 to 1980 who died by suicide during 1968–2007

There was no significant variation for season of birth in the Transition Lifestyle cohort (1951–1960) (n = 317; r = 0.04; Z = 0.45; *p* < 0.5) and no further calculations were made for this cohort.

In the Modern Lifestyle cohort, there was no significant variation for season of birth (*n* = 763; r = 0.01; Z = 0.08; *p* < 0.5). The pattern remained the same after controlling for births in the population. Figure 2b demonstrates the suicide rate per 1 000 births for those born in the Modern Lifestyle cohort for each month and Table 3 shows the average number of general births during 1961–1980, the average number of suicide births during 1961–1980 and the suicide rate per 1000 births for each month in the Modern Lifestyle cohort.

The average suicide rate was 30.4 suicides per 1 000 births. For those born in March-June, the average suicide rate was 30.8 and for those born in July-February the rate was 30.2.

## Discussion

Our finding that spring-born Inuit in Traditional Lifestyle Greenland were less suicide-prone than those born during the rest of the year is contrary to other studies [1, 2, 5, 6]. The suicide rate per 1000 births for the highest risk month (October) was 2.7 times of that of the lowest risk month (June). The seasonal variation had evened out and become slightly reversed in those born into the Modern Lifestyle. This finding raises the question of what factor in the Traditional Lifestyle spring protected the newborns from future suicide? We can only speculate.

### Light

Many researchers have proposed that light availability at the time of birth plays a role in suicide, as illustrated by the correlation between sunlight at birth and suicide victims born at that time [3]. The circadian timing system develops prenatally and matures progressively after birth [34]. Healthy preterm infants spent longer sleeping and less time feeding and gained more weight when the nursery was kept dark and quiet at night [35]. The seasonal differences in daylight are larger in Greenland than in Hungary, England and Wales [2, 5], where persons born in the spring are more suicide-prone than others and much larger than in Western Australia where no variation in season of birth was found in suicide victims [15]. If light were critical, the differences would be obvious in Greenland, due to the extreme circannual variation in natural light. It is not likely that natural light has changed much in Greenland in the 20th century, but in-door light has definitely changed a lot with the replacement of traditional oil lamps by petrol lamps and electrical light after World War II. Modern houses with glass windows also contribute to more indoor light.

### Nutrition and microbiota

Nutrition is critical for the growing foetus and infant and there have been important changes in eating habits in Greenland [36]. Long-time breastfeeding was more common in the Traditional Lifestyle [37]. Restricted foetal growth was associated with both attempted and completed suicide in the offspring in a large Swedish register study [38]. In European agricultural areas, the autumn is harvest time and usually richer in food than the spring. In Traditional Lifestyle Greenland, the spring was the best time period of nutrition since animals left their winter hibernation, which made hunting possible. Nutrition is closely related to bacterial colonisation of the intestine, which plays a major role in the post-natal development and maturation of the immune and endocrine systems and the brain. Traditional Lifestyle food promotes other microbial systems than Modern Lifestyle food. It is not a question of specific bacteria, but a lesser biodiversity in the modern life gut flora. The “biodiversity hypothesis” proposes that reduced contact with natural environmental features and biodiversity, including environmental microbiota, leads to inadequate stimulation of immune-regulatory circuits and is related to the worldwide increase in allergy and chronic inflammatory diseases [39]. There is growing evidence that the gut flora is relevant for a number of psychiatric conditions [36, 40–43], probably mediated by neuro-inflammation.

### Seasonal infections

Seasonal infections during pregnancy have been associated with serious disorders in the offspring. It has long been claimed that maternal influenza increases the risk for schizophrenia in the child, but this has not been confirmed by a recent meta-analysis [44]. Seasonal infections such as influenza would arrive with the first ships in the summer when the ice broke up and made sailing possible. The seasonal pattern of common infections was therefore quite different in Greenland compared to other countries. Today, international flights reach Greenland every day and no human habitat in Greenland is isolated for extended periods of time.

### Increased suicide rate

The massive increase in suicides from the 1970s onwards is hardly related to changes in the season of birth. Rapid societal changes have caused increasing suicide rates among *e.g.* aboriginal people adopting modern lifestyles and in Eastern Europe after the fall of communism. Loss of traditional values and the introduction of alcohol are risk factors of modern lifestyle. The total population and the life span have both increased significantly in Greenland. In modern Greenland, youth suicides have grown to be a major public health issue although the suicide rate is high among all adults [22]. In spite of the improved access to medical care, only 29 % of all suicide victims in Greenland 1968–2002 had a psychiatric diagnosis registered in their death certificate [23]. A century ago, however, almost all reported suicide cases were related to mental disorders [28]. This suggests that the underlying reasons for suicide may have changed to some extent over time. Since suicide previously was unacceptable to the Lutheran-Evangelical church, a number of accidents may have been underreported suicides. We do not know, however, if underreporting was seasonal. Every suicide is personal and the result of interaction between a number of biological and psycho-social factors. Season of birth is a biological factor, which may be overshadowed by others in the Modern Lifestyle.

### Limitations

One limitation of this work is that the population in Greenland is small and the cohorts were pooled from many years. Suicide victims who died outside Greenland were not included. Many persons born 1961–1980 are still alive and it is likely that there will be more suicides in this group after 2007.

Suicide deaths from 1934 to 50 were unavailable, but the suicide rate per 100 000 person-years was 4 in 1890–1930, 4–5 in 1924–33, 0 in 1955–59 and 14.2 in 1960–67. Since living conditions were similar, it is likely that the suicide rate was at about the same level during 1934–50. We therefore believe that unreported suicides in the Traditional Lifestyle cohort would only affect the seasonality results marginally.

Another limitation is differences in the age distribution between the cohorts. The Traditional Lifestyle cohort is older than the Modern Lifestyle cohort, which lacks cases older than 47 years of age. Age per se can be considered a risk factor for suicide, but since living conditions for all ages have changed immensely, data for one age group cannot be extrapolated to the same age group in a different time and context. Few other publications in the field have addressed age differences.

A major strength is that Greenland is one of few places where the population has lived in a Traditional Lifestyle until recently. The population is homogenous and there are reliable population statistics for most years of the study. The Rayleigh statistics used is a very sensitive method for small numbers.

## Conclusions

In conclusion, we found a highly significant variation for season of birth in suicide victims born into a Traditional Lifestyle. Those born in March-June were much less likely to commit suicide than those born at other times. This difference remained when adjusting for seasonal variation in general births. There was no significant variation in season of birth in the Modern Lifestyle cohort. Artificial light, changes in nutrition and human gut flora and seasonal infections at the time of birth may be involved; however the cause remains unknown.
